# Bioplastic Production by *Bacillus wiedmannii* AS-02 OK576278 Using Different Agricultural Wastes

**DOI:** 10.3390/microorganisms9112395

**Published:** 2021-11-21

**Authors:** Amal W. Danial, Shereen M. Hamdy, Sulaiman A. Alrumman, Sanaa M. F. Gad El-Rab, Ahmed A. M. Shoreit, Abd El-Latif Hesham

**Affiliations:** 1 Botany and Microbiology Department, Faculty of Science, Assiut University, Assiut 71516, Egypt; daniala.w@aun.edu.eg (A.W.D.); sanaafahmy@aun.edu.eg (S.M.F.G.E.-R.); 2 Pediatric Hospital, Assiut University, Assiut 71516, Egypt; Shereenhamdy80@yahoo.com; 3 Biology Department, College of Science, King Khalid University, Abha 61413, Saudi Arabia; salrumman@kku.edu.sa; 4 Genetics Department, Faculty of Agriculture, Beni-Suef University, Beni Suef 62521, Egypt

**Keywords:** Polyhydroxybutyrates (PHBs), agricultural wastes, bioplastic, bacteria, 16S rRNA analysis, NMR

## Abstract

Polyhydroxybutyrates (PHBs) are macromolecules synthesized by bacteria. Because of their fast degradability under natural environmental conditions, PHBs were selected as alternatives for the production of biodegradable plastics. Sixteen PHB-accumulating strains were selected and compared for their ability to accumulate PHB granules inside their cells. Isolate AS-02 was isolated from cattle manure and identified as *Bacillus wiedmannii* AS-02 OK576278 by means of 16S rRNA analysis. It was found to be the best producer. The optimum pH, temperature, and incubation period for the best PHB production by the isolate were 7, 35 °C, and 72 h respectively. PHB production was the best with peptone and glucose as nitrogen and carbon sources at a C/N ratio of (2:1). The strain was able to accumulate 423, 390, 249, 158, and 144 mg/L PHB when pretreated orange, mango, banana, onion peels, and rice straw were used as carbon sources, respectively. The extracted polymer was characterized by Fourier transform infrared (FTIR), nuclear magnetic resonance (NMR), and GC-MS spectroscopy, which confirmed the structure of the polymer as PHB. The isolate *B. wiedmannii* AS-02 OK576278 can be considered an excellent candidate for industrial production of PHB from agricultural wastes.

## 1. Introduction

Plastic materials that have been generally used in our daily lives are now causing dangerous environmental problems. Millions of tons of these non-degradable plastics accumulate in the environment per year. Petroleum-based plastics have serious ecological and social impacts because of their non-degradable nature and the leaching of carcinogenic substances when exposed to scratch or heat [1]. Biopolymers are one type of product that can help to overcome problems caused by petrochemical polymers. Biopolymers are generated from renewable natural sources and are often biodegradable and nontoxic. They are either produced by biological systems (microorganisms, plants, and animals) or produced from substrates obtained from living organisms such as polylactic acid, which can be synthesized from biologically obtained lactic acid [2]. Among various biodegradable polymer materials are polyhydroxyalkanoates (PHAs) [3]. PHAs are thermoplastic aliphatic polyesters with linear polymer chains that are manufactured via a microbial process on sugar-based medium, where they act as carbon and energy storage material in bacteria.

The main member of the PHAs family is polyhydroxybutyrate (PHB). It is accumulated in the cells as intracellular granules in the presence of excess carbon source or under different stress conditions, such as a limited amount of macro-components (nitrogen, phosphate, oxygen) or micro-components (sulfate, magnesium ions, and other trace elements) [4]. Bacterial PHAs could be divided into two groups depending on the number of carbon atoms in the monomeric units: short-chain-length (SCL) PHAs, which consist of 3–5 carbon atoms, and medium-chain-length (MCL) PHAs, which consist of 6–14 carbon atoms [5].

PHAs are non-toxic, biodegradable, and biocompatible, so they can be used in medical applications as bioimplant materials and in encapsulation of medicines for controlled release. It can be also used in other applications such as bags, bottles, disposable items, items of personal hygiene, films, and food packaging. PHAs degrade naturally and completely to CO_2_ and H_2_O in a natural environment due to different microorganisms [6]. Alarfaj et al. [7] proved the ability of the *B. thuringiensis* KSBM 127 strain that was isolated from the mangroves environment of Saudi Arabia to produce biodegradable plastics. The potential of isolated bacteria from the Makkah region, Saudi Arabia, to produce PHBs and the factors affecting the production of PHB were studied by Alshehrei [8].

A major problem for PHBs production is their high production cost as compared with plastics derived from petrochemicals. Therefore, much effort has been committed to reducing the production cost of PHBs by using low-cost carbon sources [9]. *Bacillus axaraqunsis* BIPC01, which was isolated from Bandar Imam, Iran, could be a potent PHB producer using petrochemical wastewater as a carbon source [10].

Some studies in Egypt were performed on PHBs isolation and characterization. Belal and Farid [11] isolated *B.cereus* from Kafr El-sheikh Governorate, Egypt, which has the ability to produce PHBs when inoculated in production media supplemented with 2% of glucose, xylose, lactose, whey, molasses, sugar cane bagasse, and rice straw hydrolysate. Rice bran, wheat bran, bagasse, cassava powder, potato starch, cassava powder, corn waste, copra oil cake, jack fruit powder, toor powder, fruit waste, and whey wastewater from industrial farming were used to form a low-cost substrate for PHBs production when inoculated with *B. cereus* [12]. Mostafa and his coworkers [13] isolated novel strains of PHB-producing bacteria (*Tamlana crocina*, *Bacillus aquimaris*, *Erythrobacter aquimaris*, and *Halomonas halophila*) from the mangrove rhizosphere, Red Sea, Saudi Arabia, which can make PHB production a decent contender for numerous industrial applications as a substitution for petroleum-based plastics. Rezk et al. (2020) [14] proved that wheat bran can be used as an alternative to starch nitrate medium for PHB production by *Streptomyces incanus* BK128.

Therefore, the aim of the present study was to optimize and characterize PHB produced by a new bacterial stain (*Bacillus wiedmanii*) with a focus on its production from some low-cost agricultural wastes (fruit peels).

## 2. Materials and Methods

### 2.1. Sample Collection and Isolation of PHB-Producing Bacteria

Different samples (soil, wastewater, sewage sludge, and cattle manure) were collected from various sources in Assiut Governorate, Egypt, and used for bacterial isolation. One gram (soil, cattle manure, or sludge samples) or 1 mL (wastewater samples) was added to 9 mL of sterilized distilled water. Samples were subjected to shaking for 30 min on a rotary shaker (150 rpm) at 30 °C. Then, serial dilutions were prepared, followed by streaking on nutrient agar plates. The plates were incubated at 35 °C for 24 h. The isolated strains were purified and maintained on nutrient slant agar and stored at 4 °C.

### 2.2. Screening the Isolates for PHB Production

The isolated strains were screened for the presence of polyhydroxybutyrate (PHB) granules using the Sudan Black B staining technique. Nutrient agar medium was supplemented with 2% glucose. The plate was divided into equal parts, and in each part, bacterial isolates were spread. The plates were incubated at 30 °C for 24 h. Sudan Black B stain was prepared by dissolution of 0.02 g powdered stain in 100 mL of 70% ethanol. After incubation, Sudan Black B dye was spread over the plates and kept undisturbed for 30 min. Plates were washed with ethanol (96%) to remove the excess stain. Colonies unable to incorporate the Sudan Black B appeared white, while PHB producers appeared bluish-black [15]. The promising isolate for PHB production was genetically identified by morphological and biochemical test and 16S rRNA gene analysis.

### 2.3. Morphological and Biochemical Analysis

The isolate was morphologically characterized by observing the standard microbiological markers (Gram reaction, motility, spore formation). The biochemical characterization of the isolate was done by series of biochemical tests including carbohydrate fermentation, H_2_S production, and catalase test. Oxidase activities and deoxidization of nitrate were also examined.

### 2.4. Molecular Identification of PHB-Accumulating Bacteria by 16S rRNA Gene Analysis

The identification was performed based on 16S rRNA gene sequence analysis. Genomic DNA was extracted from the isolated bacterium strain according to our previously described method [16]. PCR amplification was performed using common primers: 27F (5′-AGAGTTTGATCCTGGCTCAG-3′) and 1492R (5′-CGGCTACCTTGTTACGACTT-3′). The sequences obtained were then aligned with known 16S rRNA gene sequences in GenBank database using the basic local alignment search tool (BLAST) of the National Center for Biotechnology Information (http://www.ncbi.nlm.nih.gov/BLAST/, accessed on 20 September 2021). To determine the taxonomic position of the isolates, the phylogenetic tree was constructed with MEGA version 4.0 (Auckland, New Zealand) using a neighbor-joining algorithm, and the Jukes–Cantor distance estimation method with bootstrap analyses for 100 replicates was performed [17].

### 2.5. Production, Detection, and Extraction of PHB

The pure culture of the promising isolate was inoculated in 5 mL of sterile nutrient broth media. After incubation for 24 h at 30°C, 5% (*v*/*v*) of the culture was aseptically transferred into 250 mL conical flask containing 50 mL of modified mineral salts medium (pH 7.0) containing (in g/L) 20 glucose, 0.2 MgSO_4_, 0.1 NaCl, 0.5 KH_2_PO_4_, 4.0 peptone, and 2.5 yeast extract [18]. Then, it was incubated for 72 h at 30 °C and 150 rpm. The culture broth was centrifuged (MPW-260 Refrigerated Laboratory Centrifuge, MPW Med., Warsaw, Poland) at 5000 rpm for 15 min. The supernatant was discarded, and the pellet was dried. Sodium hypochlorite (10 mL) was added to the dried pellet and then incubated for 2 h at 50 °C for lyses of cells. After the incubation period, the tube with the mixture was centrifuged again at 5000 rpm for 15 min. The pellet was washed with distilled water, acetone, and methanol; then, it was dissolved by 5 mL of boiling chloroform. The non-PHB cell matter was removed by filtration using filter paper (Whatman no. 1, WHA1001045, Buckinghamshire, UK). The chloroform was evaporated, and PHB film was stored for further analysis [12].

### 2.6. Quantitative Analysis of PHB

Cell culture was grown as described earlier, and the cell pellet was dried using DZF-6020 Laboratory vacuum dry oven over night at 60 °C to estimate the dry cell weight (DCW) in units of g/L. The percentage of intracellular PHBs accumulation is estimated as the percentage composition of PHB present in the dry cell weight [15].

PHBs accumulation (%) = Dry weight of extracted PHB (g/L)/DCW (g/L) × 100.

### 2.7. Characterization of PHB

The purified PHB was characterized by the following analytical methods.

#### 2.7.1. Fourier Transform Infrared Spectroscopy (FTIR)

The instrument used for this analysis was a Nicollet 6700 FTIR spectrophotometer (Assiut University, Egypt, Thermo Fisher Scientific, 168 Third Avenue, Waltham, MA 02451, USA). PHB sample was mixed with potassium bromide (KBr) in a sufficient ratio, and then it was ground. The pellet was kept in the sample holder, and IR rays were passed through it at a range of 4000–400 cm^−1^. Obtained results were analyzed for the determination of functional groups.

#### 2.7.2. Nuclear Magnetic Resonance (NMR) Spectroscopy

The spectra were recorded for the extracted PHB sample using deuterated chloroform (CDCl_3_). Five milligrams of the extracted PHB was dissolved in deuterated chloroform (CDCl_3_), and the solution was transferred to a 5 mm NMR tube for nuclear magnetic resonance measurements. Tetramethyl saline (TMS) was used as an external reference. The instrument used for analysis was Bruker High-Performance Digital FT-NMR Spectrometer Avance (Bruker BioSpin, GmbH, Rheinstetten, Germany) with 400 MHz proton frequency, at the NMR Unit, Faculty of Pharmacy (Cairo University), and operating at the basic frequency of 400.13 MHz for ^1^H. The spectrometer is equipped with direct-detection broadband observe (BBO) probe. All NMR measurements were acquired at 298 K (25 °C). Data were analyzed using Topspin 3.1 software (Bruker Biosoin, Rheinstetten, Germany). Chemical shifts (*δ*) are expressed in ppm with reference to the residual solvent signals. Scalar coupling constants (J) are given in Hertz. The following conditions were used for recording of ^1^H NMR and ^13^C-NMR spectra: 30 °C pulse experiment; acquisition time of 4.1 s; relaxation delay 1.0 s; sweep width 15.1 ppm (8012 Hz); data points 65536; and dummy scan 2. The data were processed using line broadening 0.1 Hz.

#### 2.7.3. GC-MS Analysis

GC-MS analysis of the sample was carried out after methanolysis. PHB was suspended in 1.0 mL chloroform and 1.0 mL H_2_SO_4_/methanol (15:85) in a screw-capped tube and then heated to 100 °C for 2 h. After cooling, 0.5 mL of demineralized water was added, and the solution was vortexed for 1 min [13]. The organic phase containing the resulting methyl esters’ monomers was analyzed using Gas Chromatography-Mass Spectrometric (Varian, CP–3800 GC and Saturn 2200 MS) at Analytical Chemistry Unit, Faculty of Science, Assiut University, Egypt. 7102 Riverwood Drive, Columbia, MD 21046-2502, USA that equipped with a quadrupole ion trap mass detector coupled with CP–Sil 5 CB (0.25 mm i.d × 30 m length) capillary column. Ionization energy was 70 eV, scan interval was 1.5 s, and mass range was from 50–600 amu. The oven temperature was programmed at 50 °C for 1.0 min with 10 °C increments and held at 280 °C for 10 min. The detector and injector temperature was kept at 280 °C, and helium was used as the carrier gas.

### 2.8. Optimization of Physic-Chemical Parameters for Production of PHB

The influence of various time courses, pH, temperature, different nitrogen and carbon sources, and C/N ratio on PHB production was investigated using nutrient broth media.

#### 2.8.1. Effect of Incubation Time on PHB Production

To determine the best incubation time for PHB production, 200 mL of sterile production medium with (pH 7) was prepared and inoculated with 5% inoculums. The inoculated media was incubated at 30 °C, with shaking at 150 rpm [18]. In total, 50 mL of culture was taken periodically at 24 h intervals up to 96 h. The PHB production was determined as described previously.

#### 2.8.2. Effect of Temperature on PHB Production

To determine the optimum temperature for PHB production, 50 mL of sterile production media with (pH 7) was prepared in a different conical flask and inoculated with 5% inoculums. Each flask was incubated for 72 h at different temperatures (25, 35, and 45 °C).

#### 2.8.3. Effect of pH on PHB Production

To determine the optimum (pH) for PHB production, 50 mL of sterile production media was prepared in different conical flasks, and each flask was adjusted to different pH (4, 6, 7, 8, and 10) using 0.1 N NaOH and 0.1 N HCl. The flasks were inoculated with 5% inoculum and incubated for 72 h at 35 °C after sterilization. The PHB production was estimated as described previously.

#### 2.8.4. Effect of Different Nitrogen Sources on PHB Production

To detect the best nitrogen source for PHB production, 50 mL of sterile production media was prepared in different conical flasks supplemented with different nitrogen sources (peptone, yeast extract, urea, ammonium sulfate, and ammonium chloride) at 0.4% concentration. All flasks were inoculated with 5% of inoculum and incubated for 72 h at 35 °C after sterilization. The PHB production was estimated as described previously.

#### 2.8.5. Effect of Different Carbon Sources on PHB Production

To find out the best carbon source for PHB production, 50 mL of sterile production media containing the best nitrogen source was prepared in different conical flasks and supplemented with 2% of different carbon sources (glucose, lactose, sucrose, fructose, maltose, and starch). All flasks were inoculated with 5% of inoculum and incubated for 72 h at 35 °C after sterilization. The PHB production was estimated as described previously.

#### 2.8.6. Effect of Different Carbon to Nitrogen Ratios on PHB Production (C/N Ratio)

To detect the best C: N ratios for PHB production, the strain was inoculated in production media supplemented with different ratios of concentrations of the best C and N sources (C/N ratio as 1:1, 2:1, 4:1, 8:1, 10:1, and 20:1). The culture was incubated at 35 °C for 72 h. After incubation, PHB content was quantified according to the yields, based on which the most favorable C/N ratio was determined.

### 2.9. Development of Low-Cost Production Media Such as Fruit Peel Waste

Agricultural waste materials such as orange peel, mango peel, banana peel, onion peels, and rice straw were collected and dried for 5 to 7 days. Then, they were powdered and used for the preparation of the extract. Acid pre-treatment for agro-wastes was carried out by hydrolyzing of wastes using (0.5–5%) sulphuric acid and autoclaving wastes at 121 °C for 30 min. The extract was filtered, and the supernatant was neutralized by sodium hydroxide. The hydrolysates extract at 4% concentration, supplemented with the media component except for carbon source, were used as production media for PHB production by selected bacteria [12]. The total carbohydrate content of waste material was estimated using phenol sulphuric acid method; 0.2 mL of extract was mixed with 1.8 mL distilled water in boiling tubes. A combination of 1 mL of 5% phenol and 5 mL of 96% sulphuric acid were added into all tubes one by one and shook well so that the phenol and sulphuric acid were mixed thoroughly with working standard. After 10 min, all the tubes were placed in water bath at 25–30 °C for 15 min. Blank was set with 1 mL of distilled water, and absorbance was measured at 490 nm using a double beam spectrophotometer (Thermo Scientific, Evolution 160, Waltham, MA, USA) [19]. In addition, protein content for each type of waste was estimated according to the Lowry method using the following reagents: Reagent A, 2 g of Na_2_CO_3_ dissolved in 100 mL of 0.1 N sodium hydroxide; Reagent B, 0.5 g of CuSO_4_. H_2_O was dissolved in 1% sodium potassium tartrate. Alkaline reagent solution was freshly prepared by mixing 50 mL of reagent A with 1 mL of reagent B. Five milliliters of the alkaline reagent solution was added to 1 mL of the extract in a clean tube. Both were mixed thoroughly and allowed to stand at room temperature for at least 10 min. Then, 0.5 mL of the diluted folin reagent 1:1(*v*/*v*) was added to the above mixture and immediately mixed. After 30 min, absorbance against a blank (devoid of proteins) was measured at 570 nm with a double beam spectrophotometer (Thermo Scientific, Evolution 160, Waltham, MA, USA). A calibration curve was constructed using egg albumin [20].

### 2.10. Statistical Analysis

Statistical analysis of the data was conducted using ANOVA one-way test (analysis of variance) by SPSS program version 21 (IBM Corp., Armonk, NY, USA), and Duncan values were determined at 0.05 levels.

### 2.11. GenBank Accession Number

The nucleotide sequence of 16S rRNA gene sequences of isolated strain AS-02 reported in this study has been deposited in the DDBJ, EMBL, and GenBank nucleotide sequence databases under the name *Bacillus wiedmanni* and the accession number OK576278.

## 3. Results and Discussions

### 3.1. Screening for PHB-Producing Bacteria

PHB production by bacteria is the core subject of this work, since PHB are considered to be natural polymers. The isolated samples were screened for PHB production by Sudan Black B staining. From the obtained results, out of 30 isolates that were tested for their ability to produce PHB, 16 isolates show the ability to produce PHB. All the Sudan Black B-positive isolates were subjected to quantification of PHB production, and the best isolate was further characterized and optimized for its ability to produce a maximum amount of PHB. The PHB production was found to vary from 112–2295 mg/L, with the minimum and maximum represented by isolates AP and AS-02, respectively (Table 1). The relative PHB accumulation by the different isolates was compared to help in the identification of the best producer. Out of 16 isolates, the AS-02 strain was selected for further analysis and optimization.

### 3.2. Identification of the Isolate

PHB-producing isolate was determined on the basis of morphological and biochemical characteristics (Table 2).

**Figure 1:**
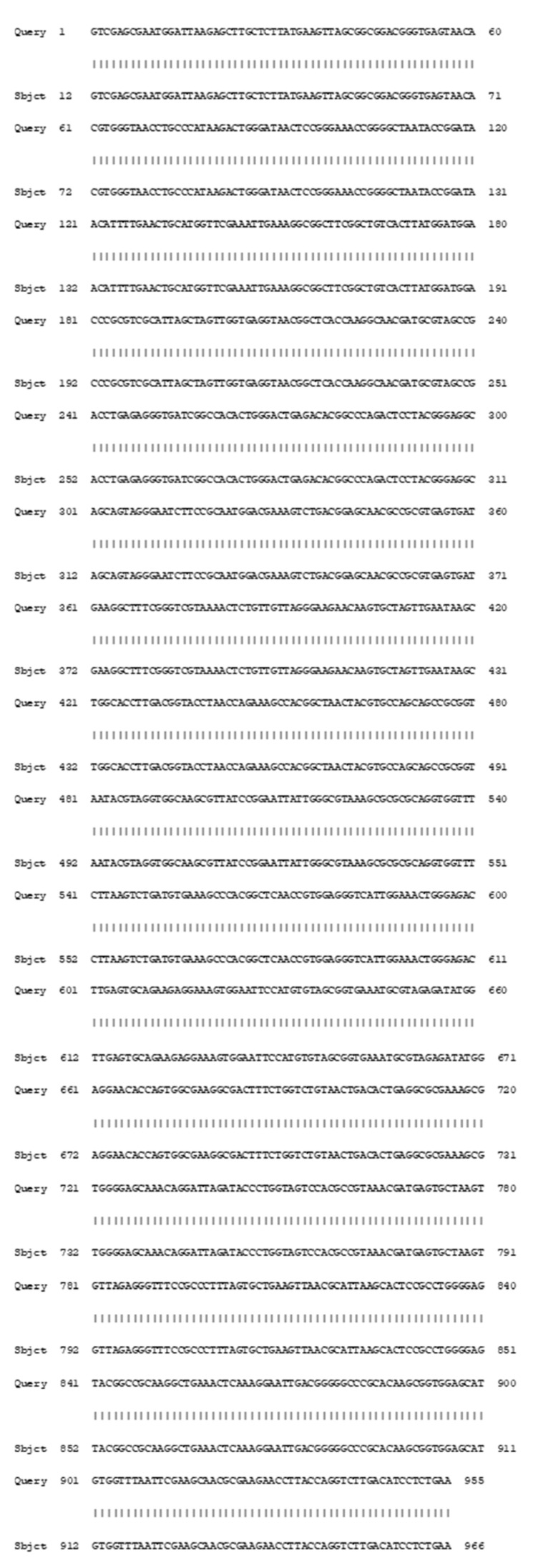
Sequence alignment of the isolate AS-02 “OK576278” (Query) against the 16S rRNA gene sequence data of *Bacillus wiedmannii* strain J5BS1 “MT415972.1” (Sbjct) in GenBank, showing NO base substitutions.

The best PHB production isolate (AS-02) was identified by comparative 16S rRNA gene-sequencing analysis. The alignment results show that the 16S rRNA sequences of the strain AS-02 were highly homologous with 100% similarities to *Bacillus wiedmanni* MT415972.1 (Figure 1).

**Figure 2:**
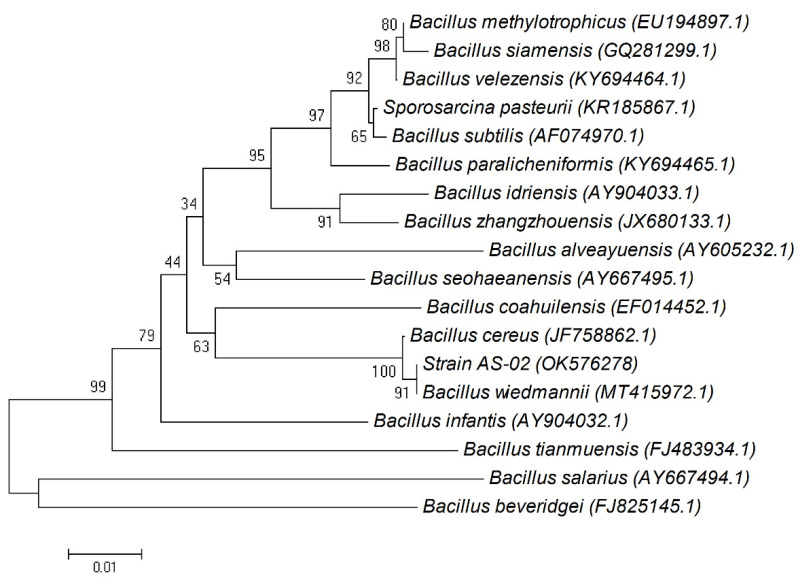
Phylogenetic tree on the basis of the patterns and the genetic relationship of *Bacillus wiedmannii* OK576278.

Phylogenetic tree indicated that AS-02 and *Bacillus wiedmanni* shared a cluster (Figure 2). Therefore, the strain AS-02 was identified as *Bacillus wiedmanni*.

The 16S rRNA gene sequences and phylogenetic analysis were considered as powerful tools for the identification of bacterial isolates [16,21,22,23].

### 3.3. Characterization of PHB

**Figure 3:**
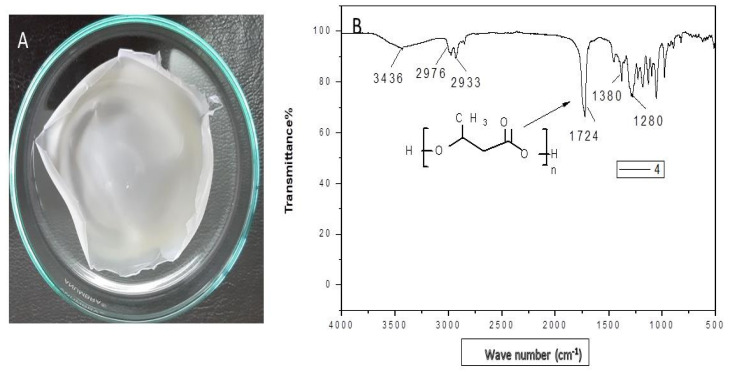
(**A**) PHB film and (**B**) FTIR analysis of PHB extracted from *Bacillus wiedmannii* strain.

The extracted polymer (Figure 3A) was characterized by Fourier transform infrared (FTIR), nuclear magnetic resonance (NMR), and GC-MS spectroscopy, which confirmed the structure of butenoic acid (the monomer of PHB).

#### 3.3.1. Fourier Transform Infrared Spectroscopy (FTIR)

FTIR spectrum (Figure 3B) obtained for the polymer extracted from *Bacillus wiedmannii* strain revealed that the band at 3436 cm^−1^ corresponds to the hydroxyl (O-H) group of alcohol, whereas the bands at 2976 and 2933 cm^−1^ are due to the methylene (C-H) stretch of alkanes. A strong peak at 1724 cm^−1^ represents carbonyl (C=O and C-O) stretch of ester. The band at 1453 cm^−1^ corresponds to CH showing asymmetrical stretching and bending vibration in CH_3_ group, whereas the band at 1380 represents the COH bond. The functional group of PHB was confirmed as C=O groups. The absorption band obtained from selected isolates was revealed to have similar peaks to standard [24], whereas the remaining peaks are closely lying between 3430 cm^−1^ to 400 cm^−1^. These absorption bands confirm the structure of PHB. The results are in agreement with the studies of other research groups working on PHB extracted from *Bacillus thuringiensis* [7].

#### 3.3.2. Nuclear Magnetic Resonance (NMR) Spectroscopy

**Figure 4:**
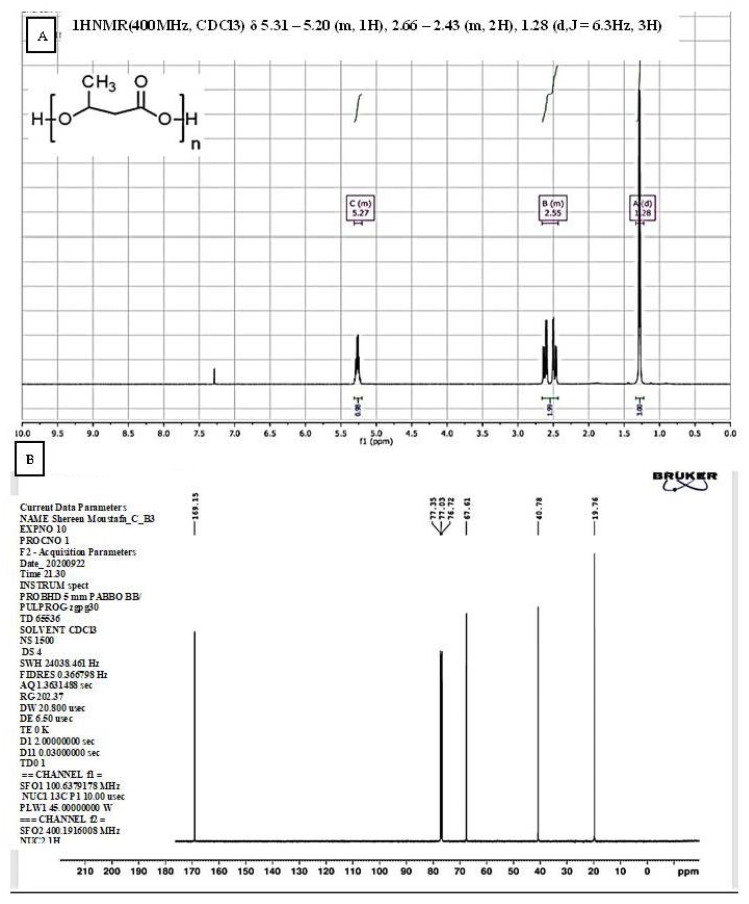
(**A**) ^1^H NMR and (**B**) ^13^C NMR spectra of polyhydroxybutyrates extracted from *Bacillus wiedmannii* strain.

The ^1^H NMR spectrum of the extracted PHB in (Figure 4A) revealed a doublet peak at 1.28 ppm, which is attributed to the methyl group coupled to one proton. The pair of quadruplets at 2.55 ppm is attributed to the methylene group (−CH_2_) adjacent to an asymmetric carbon atom bearing a single atom, and a triplet at 5.27 ppm corresponds to the (−CH) group. Another signal is observed at 7.2059 ppm, attributed to the chloroform solvent.

The ^13^C NMR spectrum of the extracted PHB (Figure 4B) shows four major peaks resembling four carbon moieties and a CDCL_3_ peak at 77.39 ppm. The signal of the quaternary carbon of the carbonyl group was detected at 169.14 ppm, while the signal of the CH group was observed at 67.59 ppm. At a high value of 40.76 ppm, the signal for the CH_2_ group appeared close to the carboxyl group, whereas the signal of the carbon of the methyl group was observed at 19.74 ppm. A similar result was observed previously by [7,25].

#### 3.3.3. GC-MS Analysis

**Figure 5:**
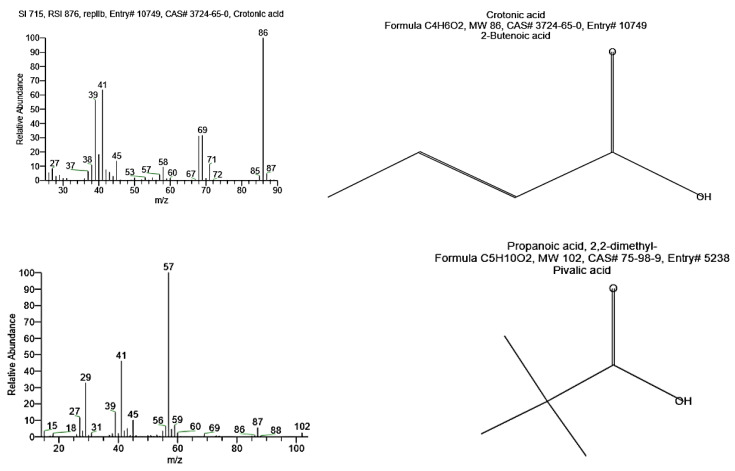

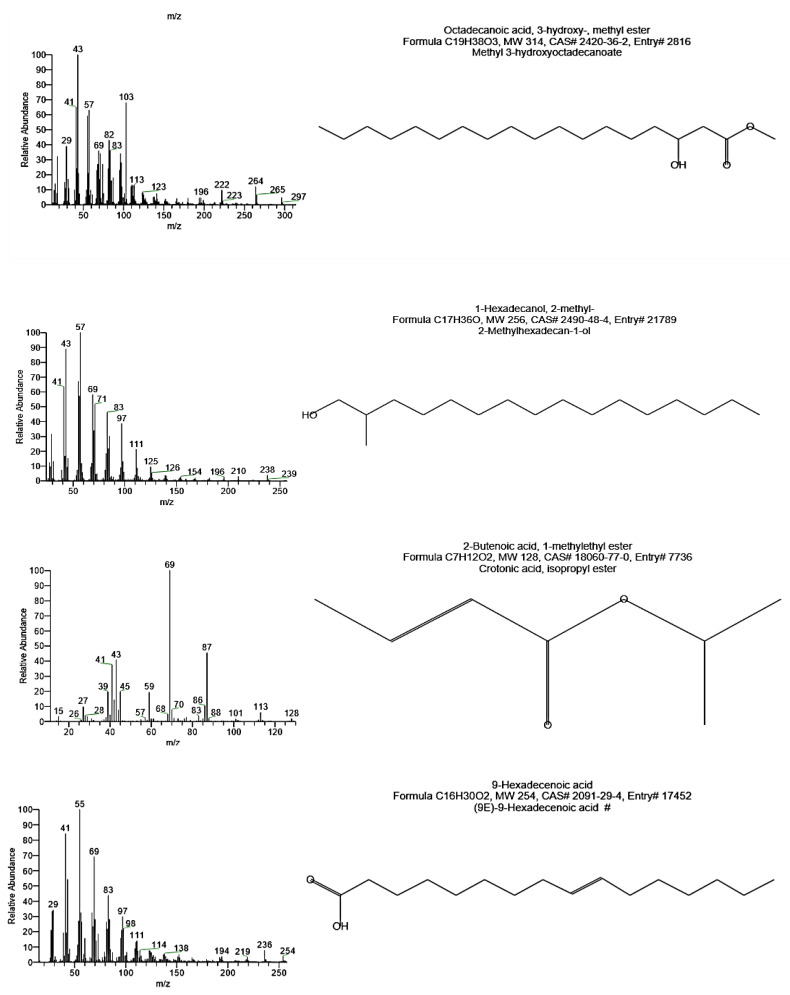
GC-mass spectra of polyhydroxybutyrate (PHB) extracted from *Bacillus wiedmannii* strain.

The PHB polymer isolated from *Bacillus wiedmannii* was analyzed by GC-MS to determine the monomeric composition of PHB (Table 3 and Figure 5). Several peaks with RT values of 4.59, 4.68, 4.9, 5.0, and 5.56 min were detected corresponding to crotonic acid, whereas another peak at RT of 17.37 is related to 2-Butenoic acid and 1-methyl ethyl ester. Additionally, a peak with RT of 7.35 correspond to propanoic acid and 2, 2-dimethyl. Multiple peaks at different RT correspond to 1-Hexadecanol, 2-methyl-, Octadecanoic acid, 3-hydroxy-, methyl ester, 1-Hexadecanol, 2-methyl-, and 9-Hexadecenoic acid, confirming the presence of PHB. These results are compatible with the previous results of Mostafa et al. [13].

### 3.4. Optimization of Cultural Parameters for Maximum PHB Production

#### 3.4.1. Effect of Incubation Time on PHB Production

**Figure 6:**
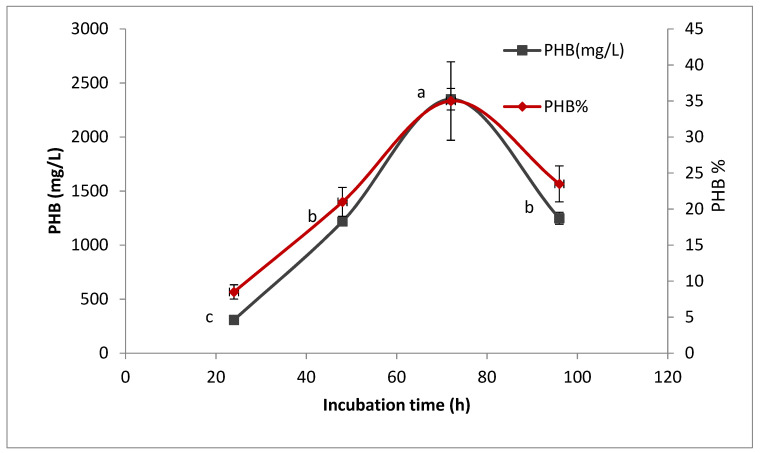
Effect of incubation time on production of polyhydroxybutyrate (PHB) by *Bacillus wiedmannii* strain. The ANOVA test was carried out by using SPSS 21 comparisons among means ± SE standard error (*n* = 3); different letters show significance at *p* = 0.05 level based on Duncan’s multiple range test.

The effect of incubation time on PHB produced by the selected isolate is shown in (Figure 6). It was found that PHB production was increased by increasing incubation time up to 72 h, after which production is decreased. This decrease in PHB production indicates that the bacteria used PHB as a nutrient source, causing unsuitable conditions due to inadequate nitrogen and carbon sources in the medium [11].

#### 3.4.2. Effect of Media pH on PHB Production

**Figure 7:**
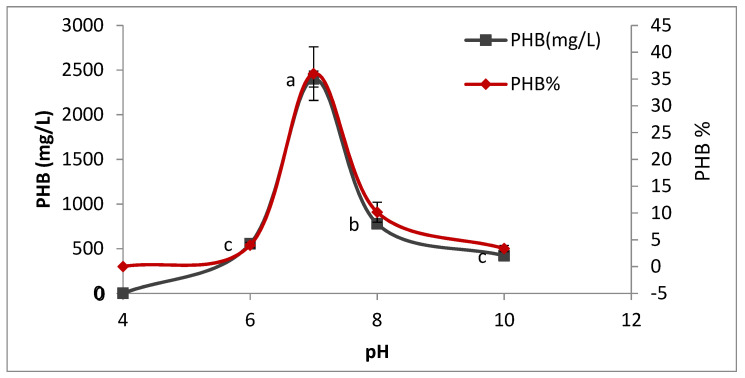
Effect of pH on the production of polyhydroxybutyrate (PHB) by *Bacillus wiedmannii* strain. The ANOVA test was carried out by using SPSS 21 comparisons among means ± SE standard error (*n* = 3); different letters show significance at *p* = 0.05 level based on Duncan’s multiple range test.

Different pH levels (4, 6, 7, 8, and 10) were checked to find out the optimum pH for PHB production. From Figure 7, it was found that the maximum PHB production 2400 ± 90 ^a^ by selected strain *Bacillus wiedmannii* is detected at pH 7. There is no PHB production at pH 4, while at pH 6 and 10, selected isolates are able to produce a low amount of PHB. This revealed that acidic and basic media are not suitable for the high production of PHB. The current observation is in agreement with earlier literature obtained by Sharma and Harish [24] and Singh [26].

#### 3.4.3. Effect of Temperatures on PHB Production

**Figure 8:**
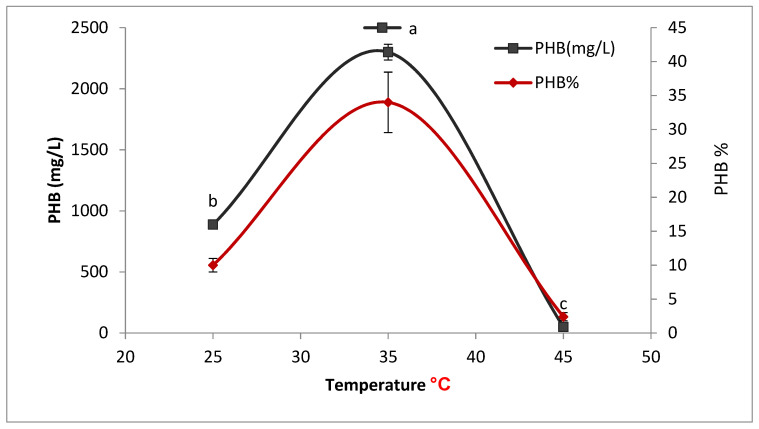
Effect of temperature on the production of polyhydroxybutyrate (PHB) by *Bacillus wiedmannii* strain. The ANOVA test was carried out by using SPSS 21 comparisons among means ± SE standard error (*n* = 3); different letters show significance at *p* = 0.05 level based on Duncan’s multiple range test.

*Bacillus wiedmannii* strain was cultivated in production media and incubated at various temperatures. It was observed that the maximum amount of PHB 2300 ± 64.5 ^a^ was produced at 35 °C (Figure 8).

#### 3.4.4. Effect of Different Nitrogen Sources on PHB Production

**Figure 9:**
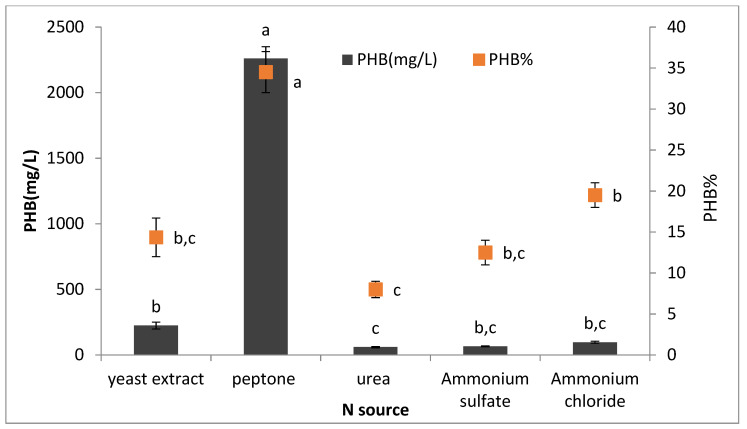
Effects of different N sources on the production of polyhydroxybutyrate (PHB) by *Bacillus wiedmannii* strain. The ANOVA test was carried out by using SPSS 21 comparisons among means ± SE standard error (*n* = 3); different letters show significance at *p* = 0.05 level based on Duncan’s multiple range test.

The effect of different nitrogen sources (yeast extract, peptone, urea, ammonium sulfate, and ammonium chloride) on PHB production was illustrated in Figure 9. *Bacillus wiedmannii* strain produced maximum PHB (2260 ± 90 mg/L) when grown in production media supplemented with peptone. It was found that complex nitrogen sources such as peptone enhanced the growth and the PHB production by *Bacillus* strain, while other nitrogen sources significantly reduced PHB production. This might be due to the relatively low nitrogen content of peptone, which in turn favored higher PHB accumulation. This result is in agreement with the previous study performed by Getachew and Woldesenbet [9].

#### 3.4.5. Effect of Different Carbon Sources on PHB Production

**Figure 10:**
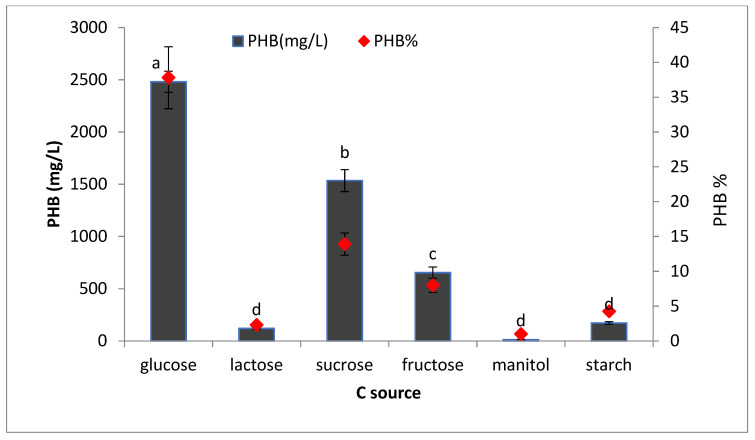
Effects of different C sources on the production of polyhydroxybutyrate (PHB) by *Bacillus wiedmannii* strain. The ANOVA test was carried out by using SPSS 21 comparisons among means ± SE standard error (*n* = 3); different letters show significance at *p* = 0.05 level based on Duncan’s multiple range test.

The data illustrated in Figure 10 show the effects of different carbon sources (glucose, lactose, sucrose, fructose, maltose, and starch) on PHB production. The results prove that the ability of the bacteria to utilize different carbon sources is variable and dependent on several factors such as the nature of the substrate used and the type of enzyme produced. It was found that glucose is the best carbon source for PHB production, followed by sucrose and fructose. Glucose is an easily useable carbon source that encourages bacteria to produce more PHB. According to the results from this study, it can be concluded that simple sugars such as glucose are easily utilized by bacteria and enhance PHB production, whereas complex molecules such as starch are not readily utilized by significant PHB production bacteria. These results are in agreement with earlier studies by Getachewand Woldesenbet [9] on *Bacillus spp* and by Sharma and Harish [24] on *Pseudochrobactrum asaccharlyticum*. From this outcome, it was observed that the maximum PHB production from glucose is 2481 ± 101.5 ^a^ for the selected isolate *Bacillus wiedmannii*. Devi et al [27] reported that PHB content in *Bacillus cereus*, reached maximum level (1.19 g/L) in a medium containing glucose (5 g/L) as carbon source. Previous literature studying PHB production under optimum condition is summarized in (Table 4) with regards to this work.

#### 3.4.6. Effect of Different Carbon to Nitrogen Ratios on PHB Production (C/N Ratio)

**Figure 11:**
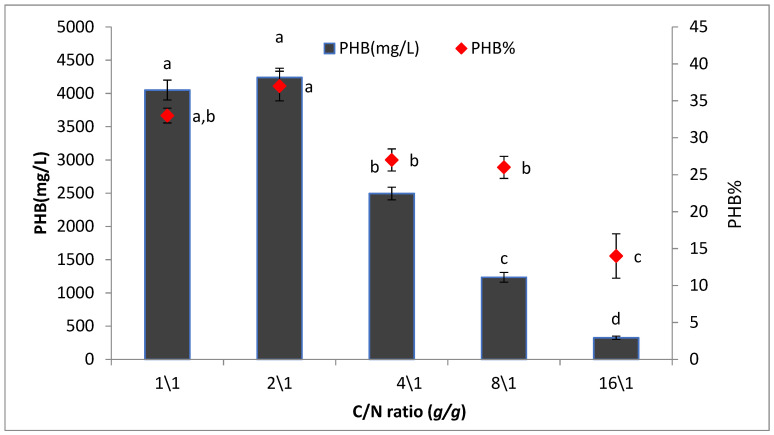
Effect of C/N ratio on the production of polyhydroxybutyrate (PHB) by *Bacillus wiedmannii* strain. The ANOVA test was carried out by using SPSS 21 comparisons among means ± SE standard error (*n* = 3); different letters show significance at *p* = 0.05 level based on Duncan’s multiple range test.

Figure 11 shows the PHB yield by *Bacillus wiedmannii* strain in the presence of different carbon to nitrogen ratio. Among the various carbons to nitrogen ratio, 2:1 was found to be the optimum carbon and nitrogen ratio supporting the maximum PHB production (4240 mg/L).

### 3.5. Production of PHB from Waste Material

There is abundant availability of waste generated in the agricultural sector, and the waste includes rich sources of carbohydrates. Species of the genus *Bacillus* have the ability to utilize these diverse and cheap carbon wastes, as they possess hydrolytic enzymes capable of metabolizing these complex residues. Different agricultural waste materials (wheat bran, rice bran, rice straw, and molasses) were employed as the main carbon source for PHB production by *Streptomyces incanus* [14], and wheat bran has the highest PHB production of 2.82 g/L. In this study, the carbon source was replaced by the extracts of five different agricultural waste materials including orange peel, banana peel, mango peel, onion peel, and rice straw for PHB production. Table 5 illustrates the total carbohydrate and protein content for each waste material.

**Figure 12:**
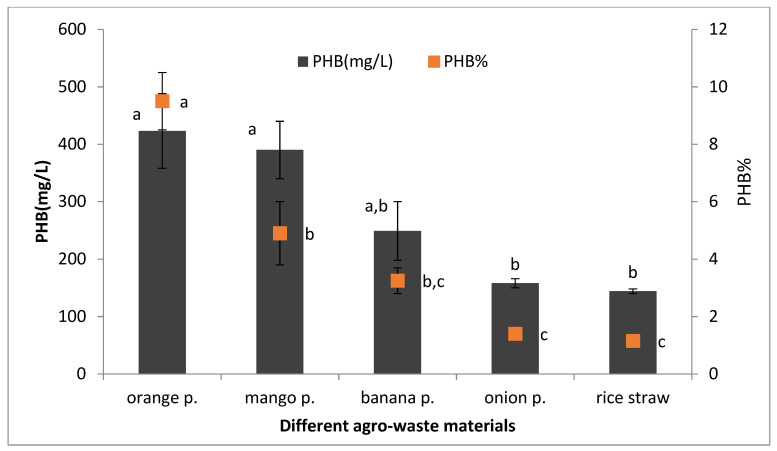
Production of PHB from agro-waste material by *Bacillus wiedmannii* strain, p. referring to peel. The ANOVA test was carried out by using SPSS 21 comparisons among means ± SE standard error (*n* = 3); different letters show significance at *p* = 0.05 level based on Duncan’s multiple range test.

These results reveal that maximum PHB production was achieved by using orange peel extract, followed by mango peel and banana peel extract, in contrast to onion peel and rice straw (Figure 12). Similarly, Gowda and Shivakumar [30] worked on the production of PHB from *Bacillus thuringiensis* IAM 12077 using agro-waste material (rice husk, wheat bran, ragi husk, jowar husk, jackfruit seed powder, mango peel, potato peel, bagasse, and straw) and obtained the highest PHB yield (4.03 g/L; 51.3%) from mango peel extract. Additionally, Sukan et al. [31] achieved the highest P(3HB) concentration of 1.24 g/L; 41% yield using orange peel as the sole carbon source in optimized medium with *B. subtilis* OK2. Table 6 shows the comparison between previous works and this study.

## 4. Conclusions

In this study, sixteen different bacterial strains were isolated and screened for PHB production using agricultural wastes as a carbon source. The isolate *Bacillus wiedmannii* from cattle manure sample at shotb area of Assiut governate was able to efficiently utilize the sugar fruit peel waste as nutrient source for PHB production. The optimum pH, temperature, and incubation period for the highest PHB production by the isolate were 7, 35 °C, and 72 h, respectively. PHB production was the best with peptone and glucose as nitrogen and carbon sources at a C/N ratio of (2:1). This study concludes that the sugar peel waste could directly serve as an inexpensive nutrient source for production of biodegradable plastic. Thus, this study may solve the problem of costly treatment of peel waste as well as high production cost of biodegradable bioplastic and help in conservation of petroleum products that are used in the commercial production of plastic.

## Figures and Tables

**Table 1:** Amount of PHB production by the different bacterial isolates. The ANOVA test was carried out by using SPSS 21 comparisons among means ± SE standard error (*n* = 3); different letters show significance at *p* = 0.05 level based on Duncan’s multiple range test.

No.	Isolates	PHB Concentration (mg/L)	PHB Content (PHB%)
1	SH	930 ± 55 ^c^	28.5 ± 2.5 ^a,b^
2	ZC1	1125 ± 25 ^c^	24.5 ± 2.5 ^c^
3	AM1	228 ± 15 ^e^	8.15 ± 1.2 ^e^
4	WS2	192 ± 7.5 ^e,f^	10 ± 1 ^e^
5	AS-02	2295 ±55 ^a^	35 ± 2 ^a^
6	MS2	560 ± 20 ^d^	21 ± 2 ^c,d^
7	EL3	162.5 ± 12.5 ^e,f^	21.6 ± 2.15 ^c,d^
8	WS1	575.6 ± 25 ^d^	23.5 ± 0.5 ^c,d^
9	MW1	180 ± 20 ^e,f^	14 ± 2 ^d,e^
10	AS-01	207.5 ± 7.5 ^e,f^	11.5 ± 1.5 ^d,e^
11	DC2	2100.4 ± 80 ^b^	28.9 ± 3.5 ^a,b^
12	DC3	1115 ± 25 ^c^	28.7 ± 1.3 ^a,b^
13	AP	160 ± 10 ^e,f^	4 ± 1 ^e^
14	EP	151 ± 6.5 ^e,f^	10 ± 1 ^e^
15	IP	247.5 ± 7.5 ^e^	4.7 ± 0.25 ^e^
16	AP	112 ± 4.5 ^f^	7.9 ± 0.1 ^e^

**Table 2:** Morphological and biochemical characterization of PHB-producing organism (AS-02 isolate). (+) positive response to the test and (−) negative response.

Test	Observation	Test	Observation
Gram stain	+	H_2_S production	−
Motility	+	Glucose fermentation	+
Spore formation	+	Sucrose fermentation	+
Catalase test	+	Mannitol fermentation	+
Oxidase test	−	Maltose fermentation	+
Urease test	+	Starch hydrolysis	+
Indole test	−	Deoxidization of nitrate	−

**Table 3:** GC-MS data table for polyhydroxybutyrate (PHB).

RT	Compound Name	Molecular Formula	Molecular Weight
4.59, 4.68, 4.9, 5.0, 5.56	Crotonic acid	C_4_H_6_O_2_	86
5.90	Propanoic acid, 2,2-dimethyl-	C_5_H_10_O_2_	102
7.41	Octadecanoic acid, 3-hydroxy-, methyl ester	C_19_H_38_O_3_	314
7.35, 7.62, 7.7, 8.4, 8.47, 8.71, 8.92, 9.0, 9.19, 9.32, 9.41, 9.72, 9.80, 11.84, 12.09, 20.2, 20.43, 25.87, 34.51	1-Hexadecanol, 2-methyl-	C_17_H_36_O	256
17.37	2-Butenoic acid, 1-methyl ethyl ester	C_7_H_12_O_2_	128
26.3, 27.04, 30.97, 34.23, 34.97, 35.46, 36.49, 37.03, 37.61, 39.5, 40.43, 41.59, 41.97, 42.24, 42.92, 43.62, 44.83, 45.42, 45.49, 45.93, 46.24, 47.01, 47.2, 48.62, 49.61, 51.15	9-Hexadecenoic acid	C_16_H_30_O_2_	254

**Table 4:** Comparison of PHB production by different bacteria under optimum condition.

Strains	Optimum Condition	PHB Concentration	Reference
*Bacillus sp*	Glucose (4%), peptone (1%), pH (7), 37 °C, and 48 h	5.0 ± 0.44 g/L	[9]
*Bacillus cereus* E6	Sucrose (2%), ammonium sulphate (1 g/L), 48 h, 30 °C, and pH 7	5.5 ± 0.2 g/L	[11]
*Pseudochrobactrum asaccharolyticum*	Glycerol, ammonium sulphate, pH (7), 30 °C, and 72 h	14.33% (*w*/*w*)	[24]
*Bacillus subtilis* NG220	Maltose(1%), ammonium sulphate (1%), 40 °C, pH (7), and 72 h	5.297 g/L	[26]
*Bacillus cereus*	Glucose (5 g/L), yeast extract (2 g/L), 48 h, and pH (7.5)	1.19 g/L	[27]
*Bacillus pasteurii*	Sucrose (2%), peptone (0.25%), 37 °C, and 48 h	36.41%	[28]
*Bacillus cereus* PW3A	Glucose (5%), peptone (0.25%), 48 h, 35 °C, and pH (7).	0.3453 g/L	[29]
*Bacillus wiedmannii* AS-02 OK576278	Glucose (2%), peptone (1%), pH (7), 35 °C, and 72 h	4.24 g/L	This study

**Table 5:** The carbohydrate and protein content of agricultural waste material.

Agricultural Waste Material	Carbohydrate Content	Protein Content
Orange peel	82.7%	8.2%
Mango peel	46.8%	6.7%
Banana peel	50.9%	6.6%
Onion peel	20.5%	9.4%
Rice straw	46.5%	4.5%

**Table 6:** Comparison of PHB production by different bacteria using different waste materials.

Strain	Waste Material as Carbon Source	PHB Concentration	Reference
*Bacillus cereus*	Whey	1.4 g/l	[11]
*Bacillus cereus*	Wheat Bran	268 (μg/mL)	[12]
*Streptomyces incanus BK128*	wheat bran	2.82 g/L	[14]
*Bacillus subtilis*	Sapota fruit peel	234 µg/mL	[25]
*Bacillus thuringiensis* IAM 12077	Mango peel	4.03 g/L; 51.3%	[30]
*Bacillus subtilis*	orange peel	1.24 g/L	[31]
*Bacillus subtilis*	Sugarcane Bagasse	258 (μg/mL)	[32]
*Bacillus wiedmannii* AS-02 OK576278	Orange peel	0.423 g/L	This study

## Data Availability

The data presented in this study are available on request from the corresponding author.
